# Special Issue “APOBECs and Virus Restriction”

**DOI:** 10.3390/v13081613

**Published:** 2021-08-15

**Authors:** Linda Chelico

**Affiliations:** Department of Biochemistry, Microbiology, and Immunology, University of Saskatchewan, Saskatoon, SA S7H 0E5, Canada; linda.chelico@usask.ca; Tel.: +1-306-966-4318

The apolipoprotein B mRNA editing enzyme, catalytic polypeptide (APOBEC) enzyme family in humans has 11 members with diverse functions in metabolism and immunity [1,2,3,4]. The enzymes deaminate cytosine in RNA or single-stranded (ss) DNA, which forms uracil. The name is derived from the first discovered family member, APOBEC1, that edits the apolipoprotein B mRNA and other mRNAs [5,6]. Uracil in RNA has a coding function, but in single-stranded (ss)DNA, it is promutagenic. Amazingly, these modification enzymes make cellular function and immunity better. For example, some family members purposefully induce these mutations in viral genomes to restrict their replication. However, events can sometimes go wrong, leading to inappropriate expression or activity, which can result in somatic mutations and cancer evolution [7,8,9]. 

The APOBEC family is divided into subfamilies that include APOBEC1, Activation Induced cytidine Deaminase (AID), APOBEC2, APOBEC4, and the APOBEC3 enzymes that are found in placental mammals [10]. In this Special Issue, the articles focus on the ability of APOBEC3 enzymes to inhibit a diverse number of viruses and act as a cross-species barrier to viruses, APOBEC3 polymorphisms, and functions of specific APOBEC3 enzymes. The articles primarily focus on how APOBEC3 enzymes inhibit human immunodeficiency virus type 1 (HIV-1), which is the virus most studied for susceptibility to APOBEC3 anti-viral activity. In this context, APOBEC3 enzymes are part of another larger family of enzymes, termed host restriction factors. It has been 19 years since the discovery of APOBEC3G-mediated restriction of HIV-1 [11]. Shortly thereafter, activities from multiple APOBEC3 family members against HIV and other viruses were discovered. There are still new discoveries being made in the APOBEC3 field, which this Special Issue summarizes in eight review articles and two research articles. 

In humans, *APOBEC3* genes are all on human Chromosome 22 and have duplicated from a single APOBEC3 gene found in placental mammals, such as mice, to seven in humans (named APOBEC3A to H, excluding E) [3]. Uriu et al. detail this evolution and discuss how the birth of the flag-ship APOBEC3, APOBEC3G, was formed [4]. The gene first occurred in Simiiformes but not in prosimians [4]. Since the birth of the *APOBEC3G* gene coincides with the invasion of endogenous retroviruses (ERVs), the evolutionary data provide strong evidence that the original function of APOBEC3G was to suppress these ERVs [4]. 

To inhibit HIV, the APOBEC3 enzymes must become encapsidated into the budding virion [2,3,12]. This enables access to the viral genome and newly synthesized (-) DNA during reverse transcription. The APOBEC3 enzymes deaminate cytosine to form uracil when the (-) DNA is single-stranded after RNaseH degradation and before (+)DNA synthesis. This results in uracil templating the addition of adenine upon synthesis of the (+)DNA, resulting in a hypermutated and likely inactivated virus. APOBEC3s can also physically block reverse transcriptase, which prevents full proviral DNA synthesis [2]. HIV produces a protein called Vif which tries to prevent APOBEC3 encapsidation by multiple mechanisms. Stupfler et al. describe how Vif uses multiple ways to block APOBEC3G activity [12]. Vif is a thermodynamically unstable protein since it is composed mainly of loops, without a stable core structure. For stability, Vif uses binding partners. The main one is the co-transcription factor CBF-β. By CBF-β binding to Vif, it is relocalized to the cytoplasm, altering the transcription profile of the cell, which includes decreasing transcription of APOBEC3G [12]. Furthermore, Vif can bind to the 5′UTR of A3G mRNA and impair translation by 70–75%. Stupfler et al. discuss how two stem-loops, SL2–SL3, in the 5′UTR are specifically bound by Vif and cause ribosome stalling [12]. Vif can also inhibit packaging of APOBEC3G through a physical interaction. Finally, the most well-known method is through Vif mediating the degradation of APOBEC3 enzymes through ubiquitination and proteasomal degradation. Vif acts as the substrate receptor in a Cullin5 ubiquitin ligase complex [12]. Nonetheless, some APOBEC3G can still escape. If Vif also becomes encapsidated, then Vif can inhibit APOBEC3G catalytic activity [12], but this does not appear to happen for other APOBEC3s, such as APOBEC3H [13]. This multifunctional role of Vif for inhibiting APOBEC3s is fascinating in itself, and Vif additionally inhibits cell cycle progression of infected cells [14]. 

In mice, the APOBEC3 (mAPOBEC3) is different from humans when restricting retroviruses. First, mice have only one *APOBEC3* gene [15]. Second, Salas-Briceno et al. describe how mouse retroviruses such as mouse mammary tumor virus (MMTV) and several strains of murine leukemia virus (MLV) are inhibited by a deamination-independent mechanism, which likely involves mAPOBEC3 binding the reverse transcriptase to block polymerase activity and increase reverse transcriptase insertion errors [15]. Additionally, during MLV infection, the mAPOBEC3 can bind directly to the protease-gag-polymerase (PR180gag-pol) precursor and perturb its autocleavage [15]. There are few to no deaminations recovered from mouse retroviruses exposed to mAPOBEC3, even with robust restriction of replication [15]. The deamination-independent mechanism has often been questioned with HIV restriction since human APOBEC3s also have strong deamination-dependent activity. However, the results in mice demonstrate how this mechanism can also be powerful. The MLV encodes an alternate glycosylated form of the Gag polyprotein that stabilizes the viral core and blocks mAPOBEC3 access to the reverse transcriptase complex [15]. The MMTV can evolve resistance to mAPOBEC3 by increasing the processivity of the reverse transcriptase [15].

All these APOBEC3 enzymes, from mice to humans, have multiple polymorphisms that affect anti-viral activity. A polymorphism in mAPOBEC3 between different strains of inbred mice led to its discovery initially as the R*fv3* gene that was either functional or not functional against suppressing the Friend virus complex (FV) [15]. In humans, with seven *APOBEC3* genes, there are numerous polymorphisms. Sadeghpour et al. describe these polymorphisms and how they affect viral infections, particularly HIV [3]. These polymorphisms are specific to certain geographical populations, likely reflecting the specialized evolution to combat viruses endemic to that area. The most striking polymorphism is a ~30 Kb deletion of all the APOBEC3B exons and introns except for the last exon (exon 8) [3]. This creates a fused APOBEC3A/B gene, which codes for a protein that is identical to APOBEC3A [3]. There are several conflicting studies on whether this deletion and other single nucleotide polymorphisms increase susceptibility to virus infection [3]. Importantly, these studies are usually done only in one geographical region, suggesting that APOBEC3 polymorphisms may not affect all populations in the same manner. 

For the APOBEC3 enzymes that restrict replication of HIV, APOBEC3G, APOBEC3F, APOBEC3H, APOBEC3D, and APOBEC3C, there are numerous polymorphisms [3]. Polymorphisms often, but not always, occur at the interaction site of the viral antagonist protein and are useful for counteracting virus suppression mechanisms [16]. Although these polymorphisms have a role in battling present day HIV infection, the origins date back to the transmission of HIV into humans from simian immunodeficiency viruses (SIV) in other primates. Uriu et al. and Gaba et al. describe how HIV was transmitted into humans [2,4]. HIV-1 is a transmission from chimpanzee or gorilla [2,4]. HIV-2 is a transmission from sooty mangabey monkeys [2,4]. Since HIV causes a lifelong infection and has been infecting primate species for millions of years, the host-pathogen interaction is highly specific. The Vif of one SIV can induce the degradation of that host’s APOBEC3 enzymes, but not other hosts [2,4]. Thus, APOBEC3 enzymes (and other restriction factors) act as cross-species barriers. Gaba et al. details how the present day amino acid sequences of HIV-1 or HIV-2 Vif were formed from adaptations made to overcome the species barrier [2]. This is also reflected in the polymorphisms of the APOBEC3 enzymes [2]. Host populations that maintain multiple polymorphisms can potentially thwart infection by “surprising” the Vif with an APOBEC3 for which it cannot induce degradation [2]. 

An important feature of most APOBEC3 enzymes that restrict HIV is their ability to multimerize, either by self-association or with RNA [1]. Multimerization on RNA promotes encapsidation of APOBEC3s into HIV virions, and for APOBEC3H specifically, dimerization with RNA is an inherent part of the enzyme structure [1]. The nature of this multimerization has been debated in the literature. Chen describes how structural studies have been able to detail these specific interactions [1]. The full-length structure of rhesus macaque APOBEC3G showed that dimerization through the N-terminal domains creates an interface for RNA to bind [1]. The full-length structure also answered the long debated question on how the two Zinc-dependent cytidine deaminase domains of APOBEC3G are orientated [1]. Although there are two cytidine domains that bind nucleic acids, only the C-terminal domain has catalytic activity. APOBEC3H is unique in that two monomers form a dimer through binding a double-stranded RNA. The implications of this unique association with RNA are discussed [1].

Foamy viruses are a unique class of retroviruses and have a unique way of avoiding APOBEC3-mediated suppression, in comparison to HIV [17]. Vasudevan et al. presents literature on foamy virus infection and APOBEC3 restriction [17]. Evidence suggests that human APOBEC3G, but not simian APOBEC3s, induces mutations, which are lethal to the virus and protect against simian foamy virus transmission [17]. Foamy viruses encode a unique protein, Bet, that is highly expressed in infected animals and was demonstrated to sequester APOBEC3s in a degradation-independent manner [17]. This results in an “immobile complex” of the APOBEC3, without triggering degradation of the APOBEC3 [17]. This interaction traps the APOBEC3s in the cytoplasm, and thus APOBEC3s are unable to become incorporated into the progeny virions. 

Although Bet appeared to use a very different mechanism than Vif to inhibit APOBEC3s, there was recently a discovery of another viral factor (BORF2) that inhibits APOBEC3s without inducing degradation [18]. Additionally, it is interesting that although the majority of studies in the field and in this Special Issue focus on APOBEC3 restriction of retroviruses, they do restrict other viruses. As described by Cheng et al., a major discovery was that APOBEC3B can restrict the replication of double-stranded DNA herpesviruses. With herpesviruses, the APOBEC3 accesses ssDNA during replication in the nucleus [19]. Evidence shows that Epstein–Barr virus (EBV), Kaposi’s sarcoma associated herpesvirus (KSHV), and herpes simplex virus-1 (HSV-1) can be targeted by APOBEC3B [19]. The initial discovery of restriction used EBV as a model system, and it was found that the ribonucleotide reductase, BORF2, binds APOBEC3B and inhibits it from accessing the viral DNA in a degradation-independent manner [19]. BORF2 caused relocalization of APOBEC3B from the nuclear compartment where herpesviruses replicate to perinuclear aggregates that co-localized with endoplasmic reticulum markers [19]. Altogether, this sets several new precedents for APOBEC3-mediated virus restriction and renews interest in searching for other viruses that APOBEC3 enzymes may inhibit.

This Special Issue also includes two research articles that provide valuable characterization of and tools for working with APOBEC3B. APOBEC3B has a somewhat ‘bad’ reputation as an APOBEC3 since it can also cause somatic mutations that contribute to cancer evolution and therapy resistance [7,8,9]. For both the anti-viral and cancer roles of APOBEC3B, more research tools, and a better structural biological understanding are needed. APOBEC3B, similar to APOBEC3G, has two Zinc-dependent deaminase domains, but only the C-terminal domain is catalytically active. Tang et al. describe the development of a high-affinity minimal antibody obtained from phage display screening that specifically binds the C-terminal domain of APOBEC3B [20]. A crystal structure of the APOBEC3B C-terminal domain in complex with the antibody was solved [20]. The antibody was found to bind to the same region as other antibodies that had been raised against APOBEC3B, suggesting that this region is highly immunogenic. The developed platform can be used to design other selective antibodies to be used as research or therapeutic tools [20]. Barzak et al. present models of the APOBEC3B C-terminal domain obtained by small-angle X-ray scattering (SAXS) [21]. The model includes analysis of an APOBEC3B C-terminal domain dimer alone and in combination with an inhibitory ssDNA oligonucleotide [21]. Interestingly, Barzak et al.’s data suggest that the APOBEC3B C-terminal domain dimer forms with a disulfide-bridge that is buried and inaccessible to reducing agents [21]. These data resulted in a model in which APOBEC3B activity could be regulated by dimerization and redox stress [21]. 

I hope that this Special Issue will serve as a resource to researchers in the APOBEC3 field. The breadth of research on APOBEC3 enzymes is overwhelming to keep up to date with for a new researcher and even for an APOBEC3 aficionado. I remember as a new postdoctoral fellow in 2004 checking PubMed daily and seeing at least five APOBEC3 articles published per month. The speed of the publications immediately after the discovery of APOBEC3G function was simultaneously marvelous and extremely daunting for a new person in the field. Although the pace has slowed down, the discoveries keep coming with new viruses that APOBEC3 enzymes restrict and more detailed knowledge of their evolution and function. These articles can surely bring anyone up to speed on the APOBEC3 field and are also a resource for all the questions still waiting to be answered. 
